# Edonerpic maleate enhances functional recovery from spinal cord injury with cortical reorganization in non-human primates

**DOI:** 10.1093/braincomms/fcaf036

**Published:** 2025-03-13

**Authors:** Koichi Uramaru, Hiroki Abe, Waki Nakajima, Wataru Ota, Michiaki Suzuki, Osamu Yokoyama, Tetsuya Yamamoto, Yukio Nishimura, Takuya Takahashi

**Affiliations:** Department of Physiology, Yokohama City University Graduate School of Medicine, Yokohama 236-0004, Japan; Department of Neurosurgery, Yokohama City University Graduate School of Medicine, Yokohama 236-0004, Japan; Department of Physiology, Yokohama City University Graduate School of Medicine, Yokohama 236-0004, Japan; Department of Physiology, Yokohama City University Graduate School of Medicine, Yokohama 236-0004, Japan; Department of Physiology, Yokohama City University Graduate School of Medicine, Yokohama 236-0004, Japan; Neural Prosthetics Project, Tokyo Metropolitan Institute of Medical Science, Tokyo 156-8506, Japan; Neural Prosthetics Project, Tokyo Metropolitan Institute of Medical Science, Tokyo 156-8506, Japan; Department of Neurosurgery, Yokohama City University Graduate School of Medicine, Yokohama 236-0004, Japan; Neural Prosthetics Project, Tokyo Metropolitan Institute of Medical Science, Tokyo 156-8506, Japan; Department of Physiology, Yokohama City University Graduate School of Medicine, Yokohama 236-0004, Japan

**Keywords:** edonerpic maleate, spinal cord injury, AMPA receptor

## Abstract

While spinal cord injury (SCI) aggravates the quality of life in humans by severe paralysis, clinical intervention to promote functional recovery from SCI is limited. We recently identified a small compound, edonerpic maleate (edonerpic MA), which accelerates training-dependent motor functional recovery from brain damage in rodents (cryo-genic cortical injury) and non-human primates (internal capsule haemorrhage) by the facilitation of experience-dependent synaptic trafficking of glutamate α-amino-3-hydroxy-5-methyl-4-isoxazolepropionic acid receptors. In the present study, we investigated whether edonerpic MA accelerates functional recovery after SCI in non-human primates. Six adult monkeys (*Macaca fuscata*) received a unilateral SCI between the C6 and C7 segment. After the SCI, upper limb motor function was immediately impaired and the animals were assigned to receive vehicle (*n* = 3) or 3 mg/kg/day edonerpic maleate (*n* = 3) by intramuscular injection for 2 months. The rehabilitative training and evaluation of behaviour using the slit task were performed 5 days a week for 2 months after SCI. The edonerpic MA-treated group showed significantly improved grasping movements than the control group. After recovery reached a plateau, we examined the somatotopic map of the contralesional primary motor cortex (M1) using intracortical microstimulation. The motor representation of wrist territory at contralesional M1 was larger in the edonerpic MA-treated group than in the control group. We concluded that edonerpic MA accelerates the recovery of grasping movements after SCI, accompanied by cortical somatotopic reorganization. Since edonerpic MA enhances recovery from damage in the central nervous system at multiple levels, treatment with edonerpic MA combined with rehabilitative training may represent a novel therapy for not only stroke but also for SCI.

## Teaser

Uramaru *et al*. revealed the efficacy of edonerpic maleate on grasping movement after cervical spinal cord injury and the change of cortical motor representation with intracortical microstimulation in non-human primates. They proposed that edonerpic maleate could be a therapeutic alternative for paralysis after spinal cord injury.

See Machado and Hollis (https://doi.org/10.1093/braincomms/fcaf076) for a scientific commentary on this article.

## Graphical Abstract

**Graphical Abstract:**
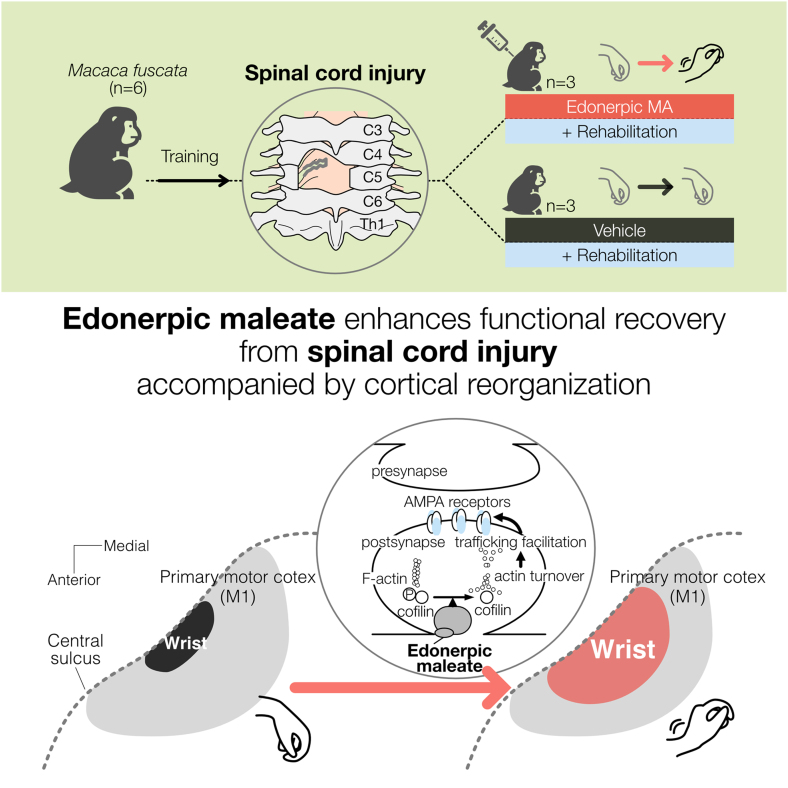

## Introduction

Around a half million people yearly suffer a spinal cord injury (SCI) worldwide.^1^ At younger ages, SCI mainly derives from acute traumatic injuries such as traffic crashes, falls or sports-related accidents; older generations primarily derive from falls or degenerative cervical spondylosis.^2^ Traumatic SCI mainly occurs at the level of cervical (−60%),^3^ which could result in limb paralysis. Despite the individual efforts of rehabilitation, SCI patients with paralysis have permanent difficulties caring for themselves and participating in socioeconomic activities.^4^

Clinical interventions to restore motor function after SCI are demanding and developing but limited in clinical practice.^4-6^ Previous animal experiments suggest cortical reorganization, cerebral activity changes or intraspinal neural rewiring accompanies motor recovery after SCI.^7-11^ Thus, accelerating these plastic changes with drugs could be a promising strategy to augment motor function in SCI-induced paralysis. The neural plasticity largely depends on the glutamate α-amino-3-hydroxy-5-methyl-4-isoxazolepropionic acid receptor (AMPAR).^12^ Glutamate AMPAR, which functions at the post-synaptic membrane, plays principal roles in excitatory neurotransmission in the brain. Trafficking of AMPAR to the post-synaptic membrane is a fundamental molecular mechanism of plastic events such as learning, memory and functional recovery from brain damage.^13-20^ We have recently identified a collapsin-response-mediator-protein 2 binding small compound, edonerpic maleate (edonerpic MA), which augments motor function recovery after brain damage in a training-dependent manner by the facilitation of synaptic trafficking of AMPAR.^20^ Administration of edonerpic MA enhances recovery of voluntary movement of the forelimb after cryo-genic cortical injury in rodents and that of the upper limb after internal capsule haemorrhage in the non-human primate.^20^

In non-human primates and humans, the cortico-spinal tracts (CSTs) are essential for finger dexterity as grasping a small object between the thumb and index fingertips.^21^ Clinical lesions in the spinal cord are not always complete transection,^22^ so sparing descending CSTs can contribute to compensation for the lost motor functions with a cortical or intraspinal circuit reorganization. Therefore, we hypothesized that edonerpic MA, a facilitator of experience-dependent synaptic AMPAR delivery to the post-synaptic membrane, would enhance the restoration from paralysis due to SCI through plastic modifications in the cortex or the sparing CSTs.

## Materials and methods

### Animals

All the experimental protocols were approved by the Committee for Tokyo Metropolitan Institute of Medical Science. Adult macaque monkeys without any history of experimentation were supplied from KAWAHARA BIRD-ANIMAL TRADING CO., LTD. and National BioResource Project ‘Japanese Macaques’ run by Kyoto University. Six adult macaque monkeys (*Macaca fuscata*, 4.6–6.4 kg, 3 years in age) were used to evaluate the efficacy of edonerpic MA on motor function recovery after induction of SCI (details about monkey’s information in Table 1). We attempted to reduce the number of monkeys to a minimum on the basis of ethical considerations and data similarity; our sample sizes were the same as those reported in previous studies by our group and others. The monkeys were housed in individual cages (width 565–700 mm, depth 730–860 mm and height 730–850 mm) under controlled conditions of temperature and light (25°C and 12-h light/dark cycle). All the experiments were conducted during the light cycle. Except for the experimental period, the monkey had access to food and water ad libitum.

**Table 1:** Demographics of the experimental animals

No.	Weight (kg)	Age (years)	Sex (male/female)	Administration	Start days of administration (POD)
Mk nm	4.6	3	Female	Edonerpic MA	5
Mk sr	5.2	3	Female	Edonerpic MA	5
Mk ym	5.3	3	Male	Edonerpic MA	7
Mean	5.0	3			5.7
Mk ht	5.0	3	Male	Vehicle	5
Mk kc	5.0	3	Female	Vehicle	5
Mk mh	5.0	3	Female	Vehicle	12
Mean	5.0	3			7.3

### Experimental time course

**Figure 1:**
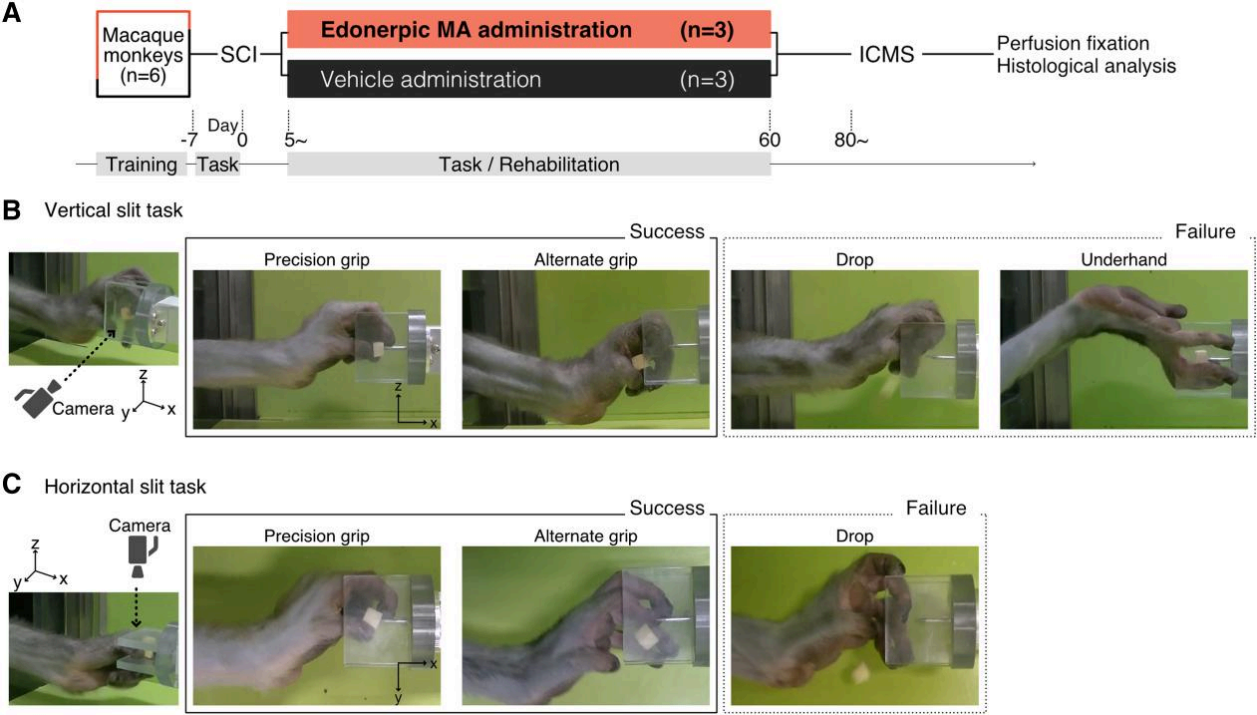
**Experimental time course and evaluation of behavioural experiments.** (**A**) Experimental time course. (**B, C**) Classification of grips and criteria for success in the vertical (**B**) and horizontal (**C**) slit task. SCI, spinal cord injury; ICMS, intracortical microstimulation.

The experimental time course is shown in Fig. 1A. The monkeys were trained to retrieve a morsel of sweet potato (∼7 × 7 × 7 mm) from a vertical and horizontal slit using the opposition of the thumb and the index finger (precision grip). The training was performed 5 days per week (lasting for 30 min to 1 h) for 2–3 months prior to SCI, and all monkeys exhibited similar baselines before SCI. After the SCI, animals were allocated to receive vehicle (*n* = 3) or edonerpic maleate (*n* = 3). All surgeons remained blind to treatment. Later than the fifth postoperative day (POD), behavioural assessment of forelimb movements was started when the monkey first reached for a food pellet using the affected hand. Since the brain concentration of edonerpic MA expected peak 15 min after intramuscular administration in previous study,^20^ rehabilitative training was started 15 min after intramuscular administration of edonerpic MA (3 mg/kg) or vehicle. We consider that days until the animal reaches their paralyzed forelimb to the task apparatus after SCI induction reflects the severity of injury. Therefore, in our current experiment, we defined the start of drug administration as the day when the animal first reached for a food pellet using the affected hand later than the fifth POD. Behavioural tasks and administration were performed 5 days per week for 56 days after SCI. After 80 days from SCI, intracortical microstimulation (ICMS) was conducted in the ipsilesional and contralesional M1 of all monkeys.

### Surgical procedure

The monkeys were first sedated using ketamine hydrochloride [10 mg/kg, intramuscular (i.m.)], xylazine (1 mg/kg, i.m.) and atropine (0.05 mg/kg, i.m.), and then anaesthesia was maintained using isoflurane (1–1.5%) inhalation. During the surgery, the following parameters were monitored: heart rate, peripheral capillary oxygen saturation, end-expiratory carbon dioxide pressure, respiration rate, body temperature and electrocardiogram. Ringer-glucose solution was administered through an intravenous (i.v.) drip. The border between the C6 and C7 segments was exposed by laminectomy of the C5–6 segments and the dura mater was cut unilaterally. After identification of the dorsal roots under a surgical microscope, the spinal cord lesion was made between the C6 and the C7 segment with watchmaker’s forceps and a special needle (27 G). The lesion was extended from the dorsal root entry zone to the ventrally. The opening of the dura mater was closed, and spongel (Astellas) was applied on the dura mater for haemostasis. The back muscles and skin were sutured with nylon or silk. Following the surgery, the monkeys were administered dexamethasone (0.25 mg/body weight/day for 3 days) and antibiotics (Viccillin; Meiji Seika, Tokyo, Japan; 40 mg/kg/day for 3 days, i.m.).

### Behavioural task

To assess functional recovery of finger dexterity before and after SCI, all the six monkeys were trained to reach and grasp task. In the task, monkeys were trained to retrieve a small morsel of sweet potato (∼7 × 7 × 7 mm) from a narrow vertical and horizontal slit (11 mm in width) using their index finger and thumb. Each testing session for the vertical and horizontal tasks consisted of 20 trials and was conducted on each experimental day. To determine the success of trial, we classified the grip into three groups based on the fingers and wrist movement during grasping: (i) index finger and thumb inside the slit (precision grip) (Fig. 1B and C); (ii) thumb outside the slit (alternate grip) (Fig. 1B and C) and (iii) wrist inverted (underhand approach, only for vertical-slit task) (Fig. 1B). A trial was considered successful when the monkey retrieved potatoes with precision or alternate grip (Fig. 1B and C) and a trial was considered unsuccessful when the monkey grasped with an underhand approach (Fig. 1B), dropped potatoes (Fig. 1B and C), used an unaffected limb and could not retrieve potatoes within a minute. The success rate was expressed as the ratio of the number of success trials to the total trials (20 trials) in each of the vertical-slit and horizontal-slit tasks.

### Administration of edonerpic MA and rehabilitation

Edonerpic MA dissolved in vehicle (15 mg/ml in 5% glucose solution) was injected intramuscularly into the lower limb. The doses were 3 mg/kg and rehabilitative training was initiated 15 min after the administration of edonerpic MA or the vehicle, based on the results of pharmacokinetic experiments as described previously.^20^ The rehabilitation session consisted of 30 trials for each of the vertical-slit and horizontal-slit tasks as in the test session.

### Electrophysiological experiments

To investigate functional neural connections from the motor cortex to the upper limbs of monkeys with SCI after rehabilitative training, we used ICMS technique. Under aseptic conditions and using isoflurane anaesthesia, limited craniotomies (20 × 20 mm) were made over the ipsilesional and contralesional M1. Rectangular plastic chambers (41 mm long × 37 mm wide × 20 mm deep) and head holders were then attached to the skull with dental acrylic. Several days later, ICMS was conducted under medetomidine anaesthesia. Ketamine was not used because of concerns about its effect on AMPA receptors. Briefly, the animals were first anaesthetized with medetomidine (30 μg/kg, i.v.) and seated in a primate chair. Their head was fixed in a stereotaxic frame attached to the chair and they received supplemental doses of medetomidine (10–15 μg mg/kg, i.v.). A glass-coated tungsten microelectrode (2.0–5.0 MΩ at 1 kHz; FHC Corporate & Manufacturing, USA) was penetrated perpendicularly to the cortical surface to a depth of 2.5–6.5 mm at intervals of 500 μm with an oil hydraulic Microdrive (MO-972A Narishige, Japan). The stimulation parameters used for ICMS were as follows: 10-pulse train, 200 μs duration at 333 Hz and current of <100 μA.

### Histological procedures

At the end of the experiments, the monkeys were sacrificed and their spinal cord was removed. The monkeys were deeply anaesthetized with an overdose of sodium pentobarbital and underwent perfusion fixation with 0.1 M phosphate-buffered saline (pH 7.3) and 4% paraformaldehyde in 0.1 M phosphate-buffered saline (pH 7.3). The spinal cord was removed immediately and saturated successively with 10, 20 and 30% sucrose solution of 0.1 M phosphate-buffered saline (pH 7.3). Serial sections of the spinal cord (50 μm thickness) were prepared from the C6/7 segment using a freezing microtome. A series of every three sections was processed for Nissl staining as follows. Images of these sections were photographed and measured the extent of spinal cord lesions using Neurolucida software (MicroBrightField, USA) and calculated the percentage of the lesioned area by the following. The gliosis area was also measured using the following Holzer staining.

#### Nissl staining

Sections were stained with 0.1% cresyl violet as described previously.^23^ Differentiation was performed in 95% ethanol containing 10% acetic acid, followed by 95 and 100% ethanol until only nuclei and Nissl bodies remained blue purple.

#### Holzer staining

Holzer staining was performed based on the website (open database) of the Digital and Computational Neuropathology of Tokyo Metropolitan Institute of Medical Science (https://pathologycenter.jp/method-e/holzer.html). Briefly, sections were immersed in phosphomolybdic alcohol, treated with a mixture of chloroform and alcohol, and stained with 2% crystal violet. They were then washed with a 10% potassium bromide solution and differentiated with aniline–xylene mixture until only the glial fibres were stained.

### Measurement of the extent of spinal cord lesions

The extent of the SCI lesion in the C6/C7 spinal cord was evaluated by the following equation: *R* = 1 − (*α*/*β*), in which *α* is the remaining area of the lesioned side, and *β* is the total area on the intact side.

### Measurement of total gliosis area around SCI

After identifying the site of maximum injury by Nissl staining, Holzer staining was performed as described above to measure the volume of gliosis in the peri-injury area of the spinal cord white matter. Twelve rostral and caudal sections were extracted at 0.5 mm intervals from the site of maximum injury and the total areas of gliosis were measured and summed to obtain the area of gliosis.

### Statistics analysis

To determine the statistical difference in behavioural analyses between the edonerpic MA-treated and control groups, a two-way ANOVA was used. *Post hoc* multiple comparisons were conducted using the Bonferroni test. For comparisons of the ICMS analyses and the extent of spinal lesions, unpaired *t*-test was applied. For comparisons of the average days from injury to the start of drug administration, ‘Mann–Whitney test’ was applied. Pearson’s product–rate correlation coefficient was used to correlate ICMS results with behavioural experimental results. Statistical analyses were conducted with GraphPad Prism 9. Cohen’s *d* values were calculated with SPSS. The statistical significance level was accepted at *P* < 0.05. In the graphs, all pooled values were represented as mean + SEM.

## Results

### The extent of spinal cord lesions between the two groups is equivalent

**Figure 2:**
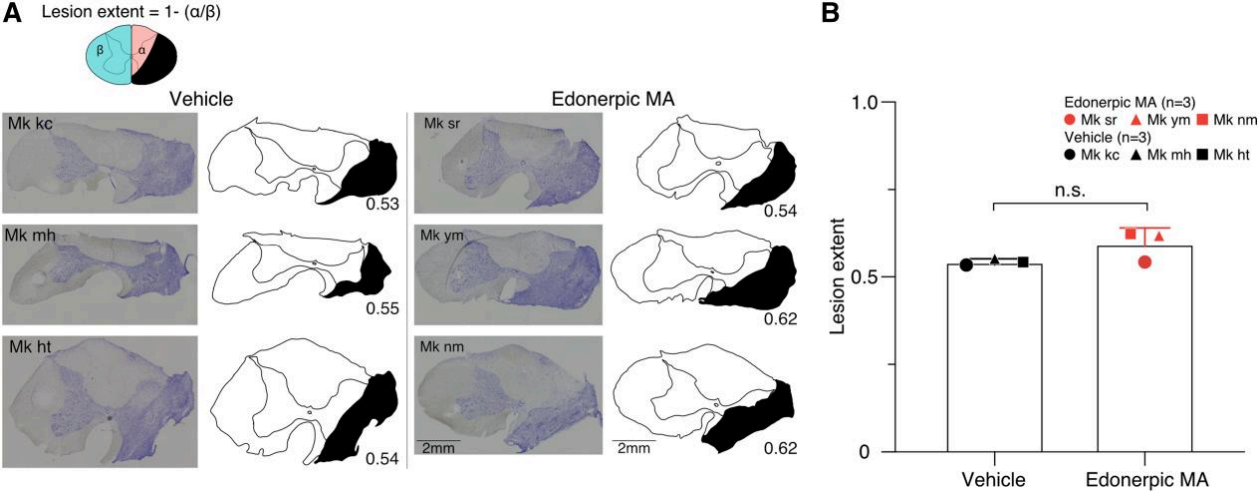
**Histological evaluation of SCI.** (**A**) Photos of Nissl staining and pictures of SCI (lesion extent in black) in each monkey. (**B**) Comparison of lesion extent in vehicle or edonerpic MA-administered monkeys. Vehicle-administrated monkeys *n* = 3 and edonerpic MA-administrated monkeys *n* = 3 (*P* = 0.13, unpaired *t*-test). Mk kc, mh, ht, sr, ym and nm represent the identification code of the monkeys. n.s., not statistically significant.

In evaluating the results of the behavioural experiments of the two groups, it is essential to confirm that there is no difference in the lesion size of the two groups. Histological analysis was performed at the end of the experiment, and the lesion size observed in the sectioned tissue was equivalent among the six monkeys (Fig. 2A); there was no statistical difference between the edonerpic MA-administered monkeys and the vehicle-administered monkeys (Fig. 2B). The lesion extent at the lateral and ventral funiculi through which the cortico-spinal tract CSTs pass was comparable between the two groups (Supplementary Fig. 1). The total extent of gliosis in the spinal cord white matter around the site of maximal injury also did not differ between the groups (Supplementary Fig. 2).

### Functional recovery improved by edonerpic MA

**Figure 3:**
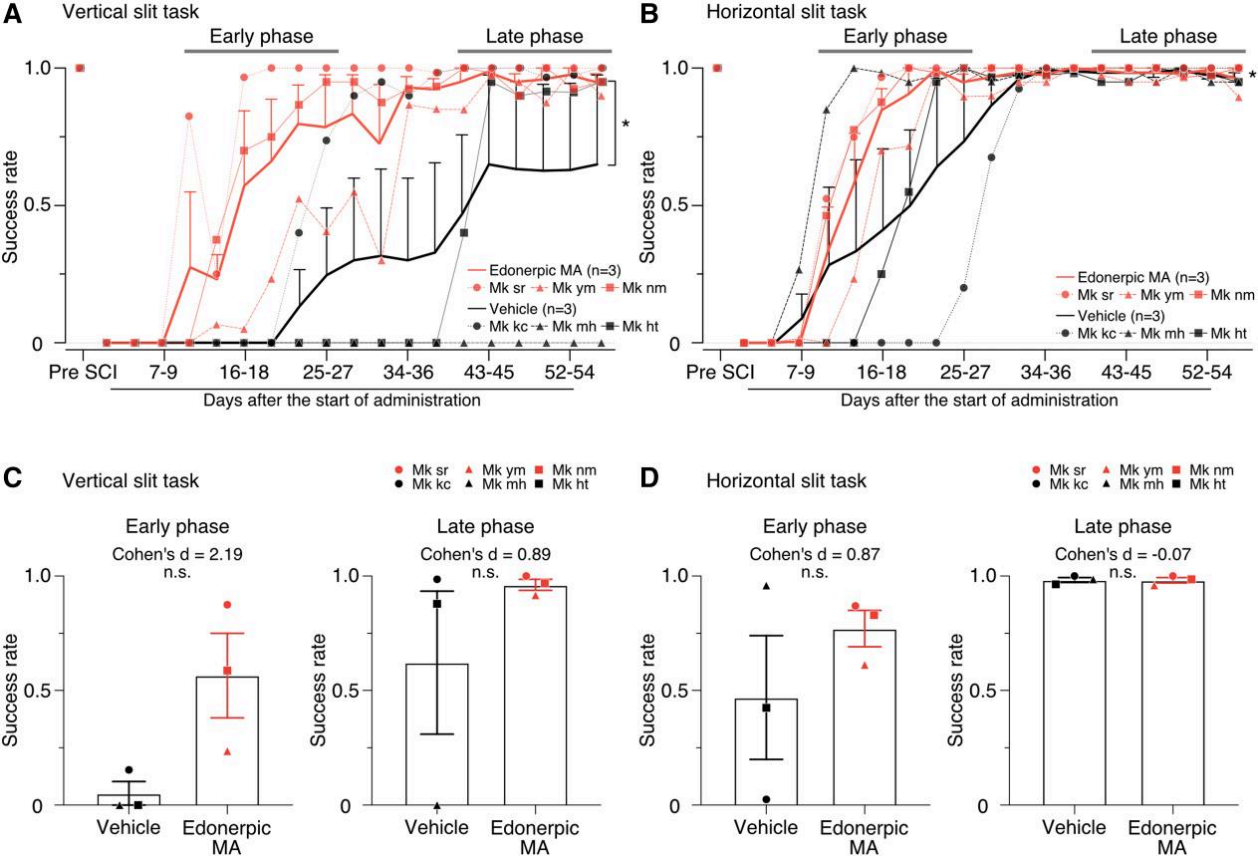
**Edonerpic MA facilitated the recovery of grasping movement after the induction of C6/7l-CSTs lesion.** (**A, B**) Changes of success rate in the vertical (**A**) and horizontal (**B**) slit task. The thin line represents each individual data, and the thick line represents the average of three monkeys. Error bars denote SEM (*P < 0.05, two-way ANOVA). (**C, D**) Comparison of average success rate in the early (Days 10–27) and late (Days 40–56) phase in the vertical (**C**) and horizontal (**D**) slit task. Each point shows the average success rate for each monkey in the early and late phase. Vehicle-administrated monkeys *n* = 3 and edonerpic MA-administrated monkeys *n* = 3 (*P* = 0.06 for the vertical-slit task in the early phase, *P* = 0.34 in the late phase, *P* = 0.35 for the horizontal-slit task in the early phase and *P* = 0.94 in the late phase, unpaired *t*-test). Mk kc, mh, ht, sr, ym and nm represent the identification code of the monkeys. n.s., not statistically significant.

The average days from injury to the start of drug administration was 5.67 days in the edonerpic MA-treated group and 7.33 days in the vehicle-treated group (Table 1). There was no statistically significant difference in these average days between the two groups (Mann–Whitney U-test). We also qualitatively evaluated the movement of the forelimb after SCI induction, which helped the judgement of group classification. Thus, we believe there is no difference in the severity of the paralyzed forelimb between these two groups. This is consistent with the finding that there is no difference in the extent of injury (Fig. 2A and B, and Supplementary Fig. 1). During longitudinal quantitative evaluations, we observed faster and more prominent recovery of finger dexterity in edonerpic MA-administered monkey than in vehicle-administered monkeys (Fig. 3A and B, Supplementary Figs 3–5 and Videos 1–3). To further explain the recovery in the early or late phase, the average success rates were compared between edonerpic MA and vehicle-administered monkeys during the early or late phase. Although we cannot show statistical significance in the comparison of edonerpic MA or vehicle-administered monkey during the early or late phase (Fig. 3C and D), Cohen’s *d* value was 2.19 for the early phase (the vertical-slit task) (Fig. 3C left), 0.89 for late phase (the vertical-slit task) (Fig. 3C right), 0.87 for the early phase (the horizontal-slit task) (Fig. 3D left) and −0.07 for the late phase (the horizontal-slit task) (Fig. 3D right). In the vertical-slit task, the vehicle-administered monkeys tried an underhand approach to the pellet (Mk mh and Mk ht in Supplementary Fig. 5A and Video 2) and the edonerpic MA-administered monkeys showed wrist dorsiflexion (Mk sr, Mk ym and Mk nm in Supplementary Fig. 5A and Video 2). In the horizontal-slit task, the vehicle-administered monkeys could not enter their thumb into the slit (Mk kc, Mk mh and Mk ht in Supplementary Fig. 5B and Video 3) and the edonerpic MA-administered monkeys could re-build the nearly former precision grip using their index finger and thumb (Mk sr, Mk ym and Mk nm in Supplementary Fig. 5B and Video 3).

### Edonerpic MA causes cortical reorganization that contributes to dexterity

**Figure 4:**
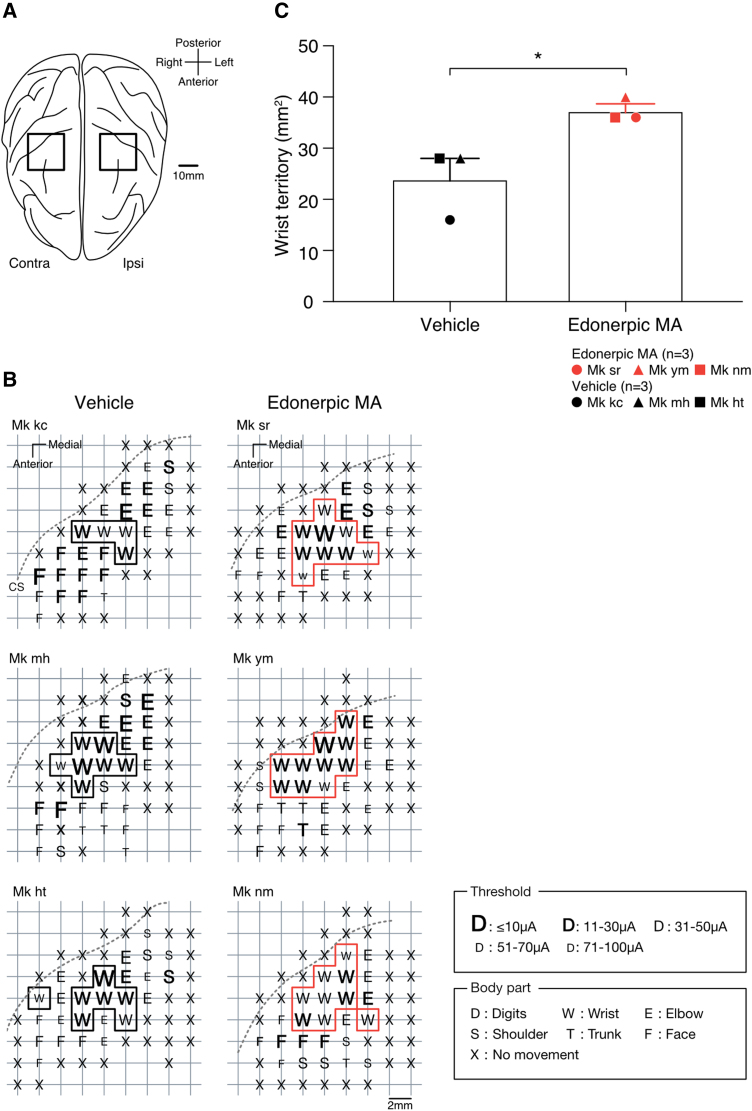
**Edonerpic MA broadened the somatic localization of the wrist.** (**A**) ICMS sites around M1 (square) in both hemispheres. (**B**) Results of ICMS mapping of contralesional hemisphere. At each penetration point, the movement triggered by the lowest threshold is recorded as the body part representing that point. (**C**) Comparison of wrist territory on the contralesional hemisphere. Vehicle-administrated monkeys *n* = 3 and edonerpic MA-administrated monkeys *n* = 3 (*P = 0.03, unpaired *t*-test). CS, central sulcus. Mk kc, mh, ht, sr, ym and nm represent the identification code of the monkeys.

Following behavioural assessment, these six monkeys were examined for somatotopy in both hemispheres around M1 (primary motor cortex) with ICMS (Fig. 4A). In the ICMS protocol, we placed stimulating electrodes in the contra and ipsilesional hemisphere. The somatotopic map was characterized based on the movements of digits, wrist, elbow, shoulder, trunk (including leg) or face in response to the stimulation via the electrodes (Fig. 4B). Specifically, when stimulating, we checked the movement on both the impaired and the unaffected forelimbs. Only movements of the affected side were detected for stimuli in the contralesional hemisphere and only movements of the unaffected side were detected for stimuli in the ipsilesional hemisphere. In addition, we did not detect any territory responsible for moving the digits in both groups of monkeys when stimulating the contralesional hemisphere under the stimulating threshold. After completing the somatotopic mapping, the number of mapping points corresponding to the wrist movements was measured. Each responsive site represented 4 mm^2^ (2 mm × 2 mm) of cortical surface, from which wrist territory was calculated. Edonerpic MA-administered monkeys had a significantly wider wrist territory on the contralesional hemisphere than vehicle-administered monkeys (Fig. 4C). There was no statistical difference between these two groups in the wrist territory on the ipsilesional hemisphere (Supplementary Figs 6 and 7). Further, we did not detect the statistical significance of the territories in other movements than wrist (digits, elbow and shoulder) in both ipsi and contralesional hemispheres (Supplementary Figs 6 and 7). In addition, although there was no statistical significance, there was a negative correlation between the area of the wrist territory and the number of days until the success rate recovered to 50% or more (Supplementary Fig. 8).

## Discussion

The main finding of this study was that administration of edonerpic MA and combined rehabilitation accelerated the recovery of grasping movements after SCI, accompanied by cortical somatotopic reorganization. This is probably due to the rehabilitation-dependent augmentation of AMPA receptor trafficking to post-synaptic membranes by edonerpic MA as we previously demonstrated.^20^

We transected between C6 and C7 segments of spinal cord. The lesion area of our C6–7 lesion involved not only the lateral funiculus but also a part of anterior (ventral) funiculus (Fig. 2A). While the spontaneous grasping recovery is observed around 40 days after C4–5 transection in the vertical-slit task in a previous report,^8^ the insufficient recovery was observed even after 40 days in our present study in the vertical-slit task. It is inferred that the broader injured area of spinal funiculus would yield the more severe paralysis, which might demand more time for spontaneous recovery.

We showed in the previous report that plasma and brain concentrations of orally administered edonerpic MA move in parallel and that brain concentration is higher than plasma concentration in mice, which means that edonerpic MA exhibits a high CNS penetration.^20^ We also confirmed that the plasma concentration of edonerpic MA reaches the peak 15 min after intramuscular injection in the non-human primate. Edonerpic MA is considered to have high brain permeability and CNS concentrations expected to peak 15 min after intramuscular administration.^20^ In our previous study when we evaluated the rehabilitation-accelerating effects of edonerpic MA in an internal capsule haemorrhage model, we started rehabilitation 15 min after intramuscular administration, demonstrating a significant enhancement of the rehabilitation effect by edonerpic MA.^20^ Based on these results, we considered that initiation of rehabilitation 15 min after intramuscular administration of edonerpic MA would be beneficial in non-human primates SCI model.

We consider that days until the animal reaches their paralyzed forelimb to the task apparatus after SCI induction reflects the severity of injury. Therefore, in our current experiment, we defined the start of drug administration as the day when the animal first reached for a food pellet using the affected hand later than the fifth POD. As a result, the average days from injury to the start of drug administration was 5.67 days in the edonerpic MA-treated group and 7.33 days in the vehicle-treated group as you can confirm in the Table 1. There was no statistically significant difference in these average days between the two groups (Mann–Whitney U-test). We also qualitatively evaluated the movement of the forelimb after SCI induction, which helped the judgement of group classification. Thus, we believe there is no difference in the severity of the paralyzed forelimb between these two groups. This is consistent with the finding that there is no difference in the extent of injury (Fig. 2A and Supplementary Fig. 2).

**Figure 5:**
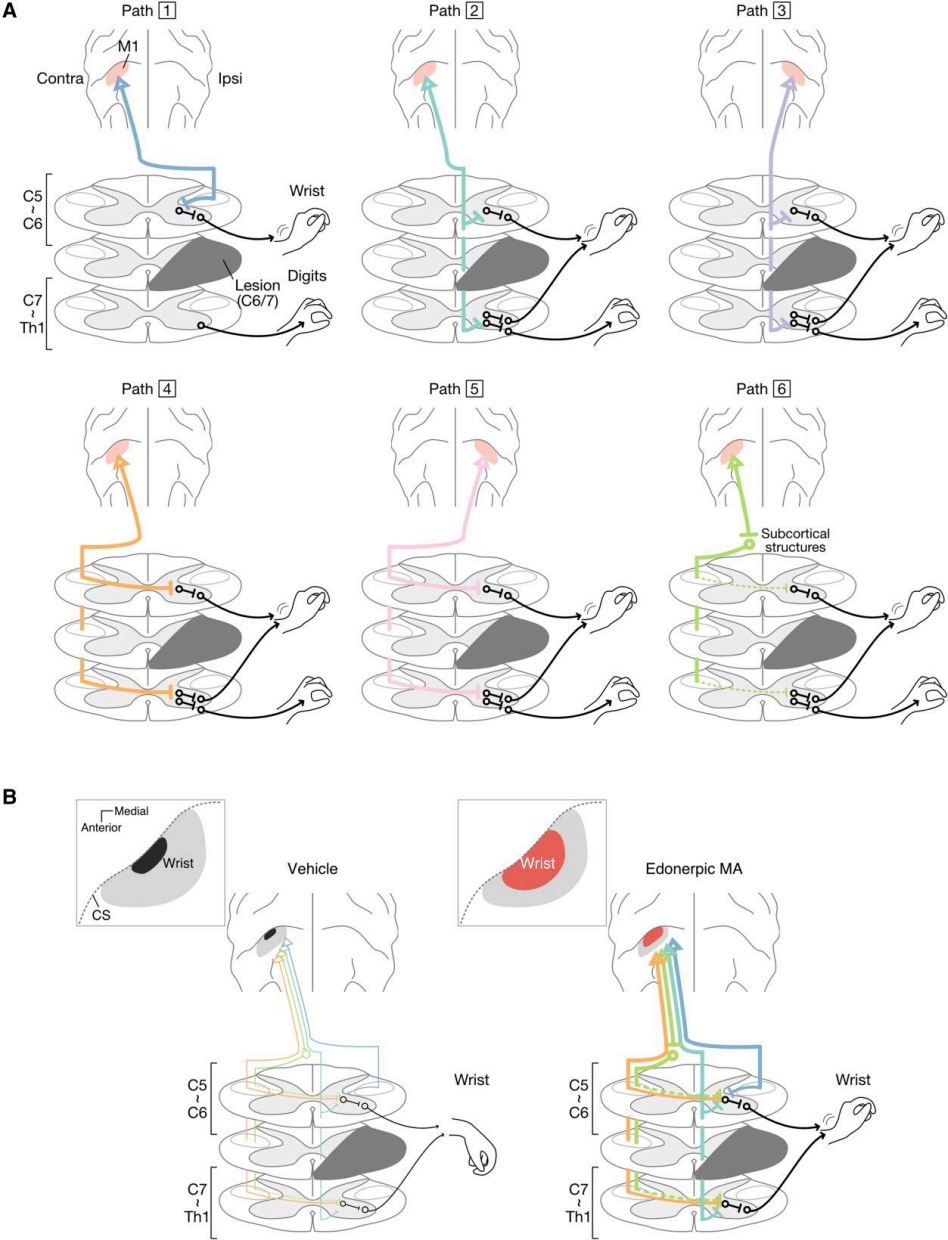
**Schema of cortico-spinal pathways contributing to the recovery with edonerpic MA after SCI.** (**A**) Residual pathways after induction of C6/7l-CSTs lesion. (**B**) Cortico-spinal pathways proven to increase synaptic efficiency in our ICMS and presumed to contribute to the process of recovery. CS, central sulcus.

Our previous report shows that edonerpic MA promotes the motor function recovery by facilitating the AMPA receptors trafficking to the post-synaptic membrane in the injury-spared regions in a rehabilitation-dependent manner.^20^ As shown in Fig. 5, the recovery after C6–7 transection augmented by edonerpic MA is thought to be achieved through the enhanced AMPA receptors trafficking to the post-synaptic membranes at the synapses involved in residual cortico-spinal Pathways 1, 2, 4 and 6. These cortico-spinal pathways have been shown to exist prior to the induction of C6–7 transection.^23^ Edonerpic MA does not form new circuits through axonal regeneration, but strengths the functional connection between neurons of the residual pathways. Therefore, effects of edonerpic MA can be observed early after administration (Fig. 3).

It is well known that SCI could lead to detrimental changes, which include abnormal proprioceptive reflex responses,^24,25^ increased muscle tone,^26^ spontaneous spasms,^25^ bladder overactivity,^27^ neuropathic pain^28^ and autonomic dysreflexia.^29^ These maladaptive changes are derived from reorganization failures in the remaining and damaged neurons in the spine after complete SCI. Maladaptive plasticity is more prevalent in cases of complete SCI compared to incomplete SCI. After incomplete transection of the spine, spontaneous recovery depends on the spared descending pyramidal tracts.^30,31^ In the present study, we induced an incomplete transection of the spine so that the vehicle group might show spontaneous recovery rather than maladaptation. We did not observe spasms or muscle tone abnormality in the forelimb both in the vehicle and edonerpic MA groups during the evaluation period. The combination of edonerpic MA and rehabilitation for a paralyzed upper limb could not cause maladaptive plasticity because we consider that edonerpic MA can induce on-demand plastic changes in a rehabilitation-dependent manner.^20^

Wrist movements are controlled by the motoneurons projecting the upper limb muscle, which positions from C5 to Th1. These motoneurons have projections dominantly from l-CST descending from the contralesional M1, crossing in the medulla oblongata and descending the dorsolateral funiculus of ipsilesional side. After SCI, this pathway is mainly disconnected and limb motor function was immediately impaired but partially spared at the rostral level of lesion (Path 1 in Fig. 5A). In addition, since the extent of SCI does not include a portion of the ventromedial funiculus, the CST passing through this area is also spared^23,32^ (Paths 2 and 3 in Fig. 5A). On the other hand, there are collateral pathways from l-CST descending the contralesional side and crossing at the spinal level (Paths 4 and 5 in Fig. 5A) and via subcortical structures tract, e.g. the rubrospinal tract (Path 6 in Fig. 5A). These residual pathways may contribute to recovery from SCI. It is considered that biotinylated dextran amines tracing of neural fibre in descending pyramidal tract^33^ or non-invasive spinal tractography with diffusion tensor imaging^34^ are useful for elucidating the neural substrates underlying edonerpic MA’s therapeutic effects.

It is known that there is a correlation between recovery after SCI and cortical representations detected by ICMS.^35,36^ In the present experiment, the edonerpic MA-administered monkeys showed significantly improved fine motor skills and larger wrist territory than the vehicle-administered group in the contralesional hemisphere. These results suggest that edonerpic MA contributes to the recovery from SCI by increasing the functional connectivity of the residual pathway and cortical reorganization involved in the movement of the paralyzed limb motor function. In addition, although it did not reach statistical significance, there was a negative correlation between the wrist territory and the number of days to recovery, and the fact that the wider the wrist territory, the shorter the number of days to recovery, also strengthens this suggestion.

The residual pathways (Paths 2–6 in Fig. 5A) also contribute to digit movement recovery and edonerpic MA may increase functional connectivity of these pathways and further accelerate recovery. However, due to the small number of residual pathways, we did not detect any territory responsible for moving the affected digits in both groups under the stimulating threshold.

On the other hand, the wrist territory expansion, demonstrated using ICMS in this experiment, may also contribute to the recovery of fine motor skills. This may be due to tenodesis action, which is widely known as compensatory dorsiflexion of the wrist supporting grasp movement after SCI.^37,38^ Edonerpic MA-administered monkeys could dorsiflex wrists and retrieve potatoes as pre-SCI, whereas the vehicle-administered monkeys could not dorsiflex wrists against gravity and tried the underhand approach (Video 2 and Supplementary Fig. 5). It is thought that tenodesis action exists behind the recovery and that the administration of edonerpic MA strengthened functional connectivity responsible for wrist movement and contributed to the prominent recovery. The significantly larger wrist territory is consistent with the results of the behavioural experiments.

The main limitation of this study is its small sample size. However, in the present study, the underhand approach was observed only in the control group, whereas in the edonerpic MA group, retrieve was observed with the same approach as before SCI. These clear results, as well as the results of a previous study using edonerpic MA in a primate cerebral haemorrhage model,^20^ explain the validity of conducting this study with two to three animals per group. Due to the sample size limitation, statistical significance cannot be demonstrated in Fig. 3C and D. However, Cohen’s *d* values, 2.19 for the early phase (Fig. 3C left), 0.89 for the late phase in the vertical-slit task (Fig. 3C right) and 0.87 for the early phase of the horizontal-slit task (Fig. 3D left) explain better recovery in the edonerpic MA-administered monkeys than the vehicle-administered monkeys because Cohen’s *d* value is 0.8 or greater, indicating a large effect size.^39,40^ The purpose of additional analyses (Fig. 3C and D) was to more visually demonstrate that the edonerpic MA-administered monkeys recovered from the early phase better than the vehicle-administered monkeys.

The lack of necessary nerve tracing is one of the limitations of this study. This is because we prioritized electrophysiological experiments to prove our hypothesis that edonerpic MA promotes functional recovery by increasing synaptic transmission efficiency after SCI. Another limitation is that it is unclear whether the full recovery could occur or not in the control group in the vertical-slit task because we did not test the later time points. In fact, we observed the success rate after around 43 days reached plateau in the vertical-slit task (Fig. 3A) in the vehicle and drug groups, thus the edonerpic MA could induce the overall improvement in the vertical-slit task.

In our previous study,^20^ we demonstrated that edonerpic MA accelerated functional recovery of motor function from the cortical cryoinjury (rodent) and internal capsule haemorrhage (macaque monkey) by the facilitation of experience-dependent synaptic AMPAR delivery. In the current study, we further proved that edonerpic MA improved functional recovery even after SCI by augmented cortical somatotopic reorganization. Therefore, edonerpic MA can promote recovery from paralysis induced by the injury at distinct areas in the pyramidal tract that includes the cortex, the internal capsule and the spinal cord, indicating that edonerpic MA can be effective to enhance motor functional recovery from the damage throughout the pyramidal tract.

## Supplementary Material

fcaf036_Supplementary_Data

## Data Availability

The original data that support the findings of this study are available in the supplementary information. The investigators were not blinded, but all the experimental data were stored on video and can be disclosed upon request.
