# Supplementary material for: Edonerpic maleate enhances functional recovery from spinal cord injury with cortical reorganization in non-human primates
Source: Brain Commun. 2025 Mar 13;7(2):fcaf036. doi: 10.1093/braincomms/fcaf036 (PMC11997772; doi:10.1093/braincomms/fcaf036)
Supplement: fcaf036_Supplementary_Data [file fcaf036_Supplementary_Data.zip › Supplementary_Figures.pdf]

Supplementary Material

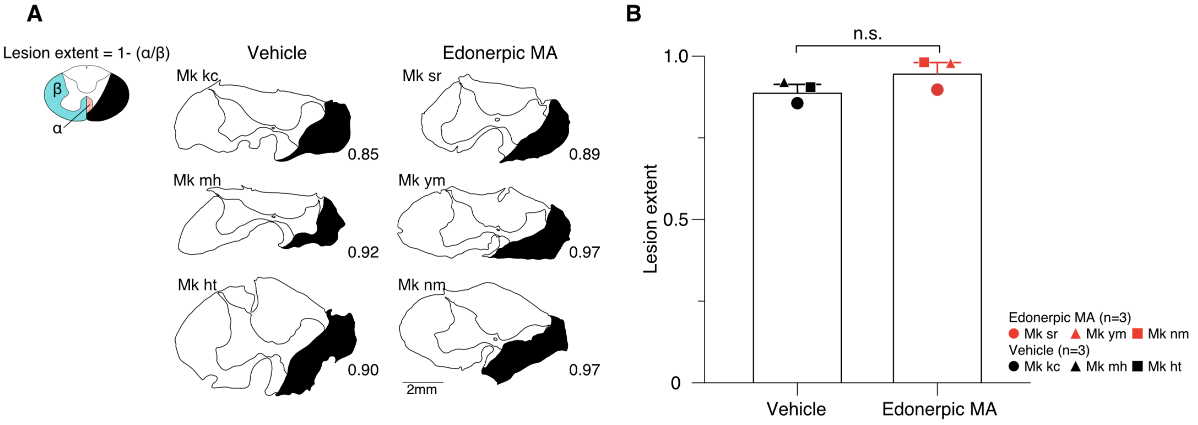

Supplementary Figure 1 Lesion extent at the lateral and ventral funiculi. (A) The lesion size observed in the sectioned tissue of the six monkeys (overlap with Figure 1), and lesion extent at the lateral and ventral funiculi. **(B)** There was no statistical difference in lesion extent between the edonerpic MA-administered monkeys (N = 3) and the vehicle-administered monkeys (N = 3) (P = 0.15, unpaired t-test). Mk kc, mh, ht, sr, ym, and nm represent the identification code of the monkeys. n.s.; not statistically significant.

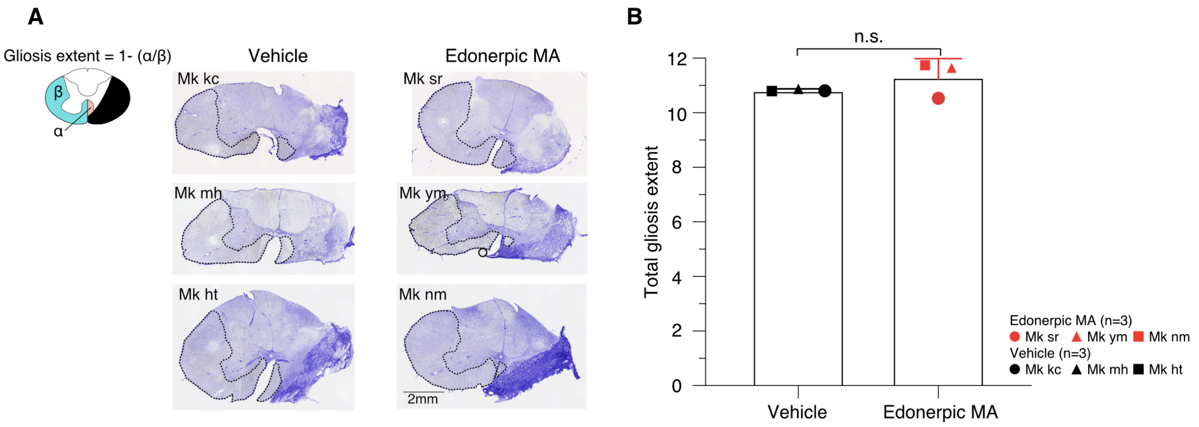

Supplementary Figure 2 Total gliosis extent at the lateral and ventral funiculi. (A) The extent of gliosis observed in representative tissue sections (Holzer staining) of six monkeys, and gliosis extent at the lateral and ventral funiculi (dotted lines represent peri-injury area of the spinal cord white matter). **(B)** There was no statistical difference in total gliosis extent between the edonerpic MA-administered monkeys (N = 3) and the vehicle-administered monkeys (N = 3) ($P = 0.29$, unpaired t-test). Mk kc, mh, ht, sr, ym, and nm represent the identification code of the monkeys. n.s.; not statistically significant.

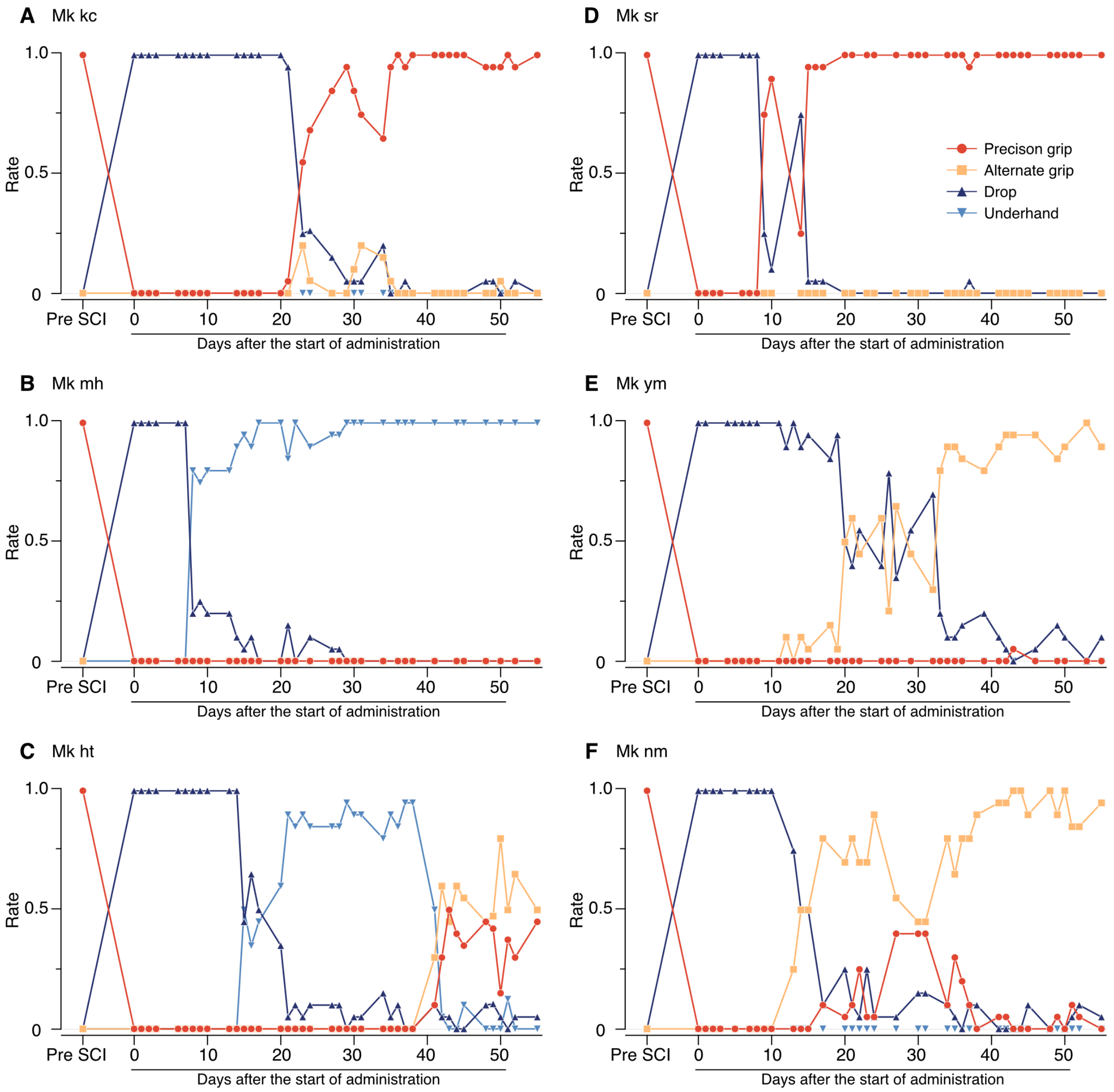

Supplementary Figure 3 Time course of results for the vertical slit task in each monkey. (A-C) Results from vehicle-administered monkeys (Mk kc, mh, and ht). **(D-F)** Results from edonerpic MA-administered monkeys (Mk sr, ym, and nm).

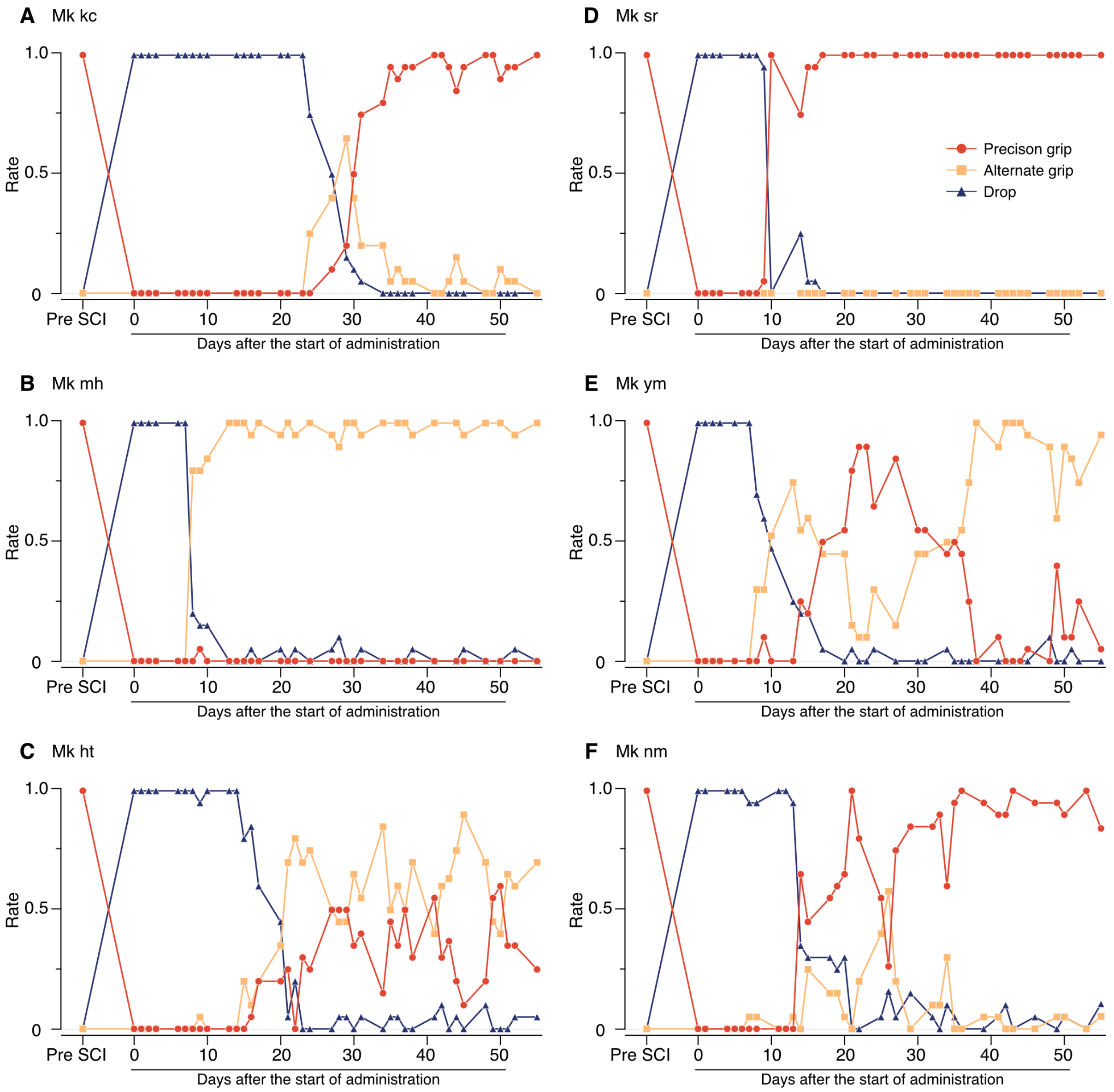

Supplementary Figure 4 Time course of results for the horizontal slit task in each monkey. (A-C) Results from vehicle-administered monkeys (Mk kc, mh, and ht). **(D-F)** Results from edonerpic MA-administered monkeys (Mk sr, ym, and nm).

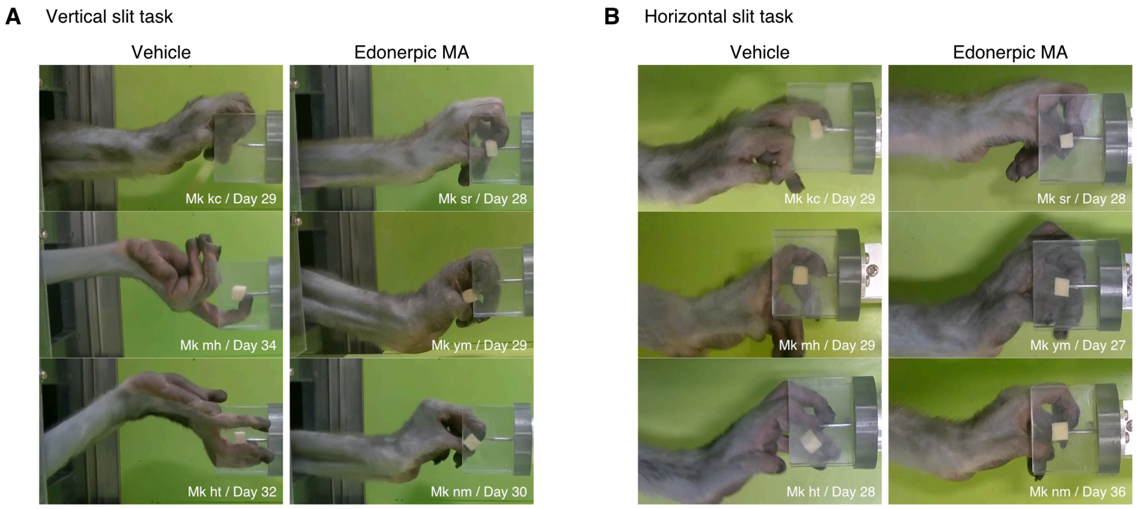

Supplementary Figure 5 Representative approaches to the pellet after SCI. (A) Vertical slit task. **(B)** Horizontal slit task. Mk kc, mh, ht, sr, ym, and nm represent the identification code of the monkeys. Days after the start of administration are shown in each snapshot.

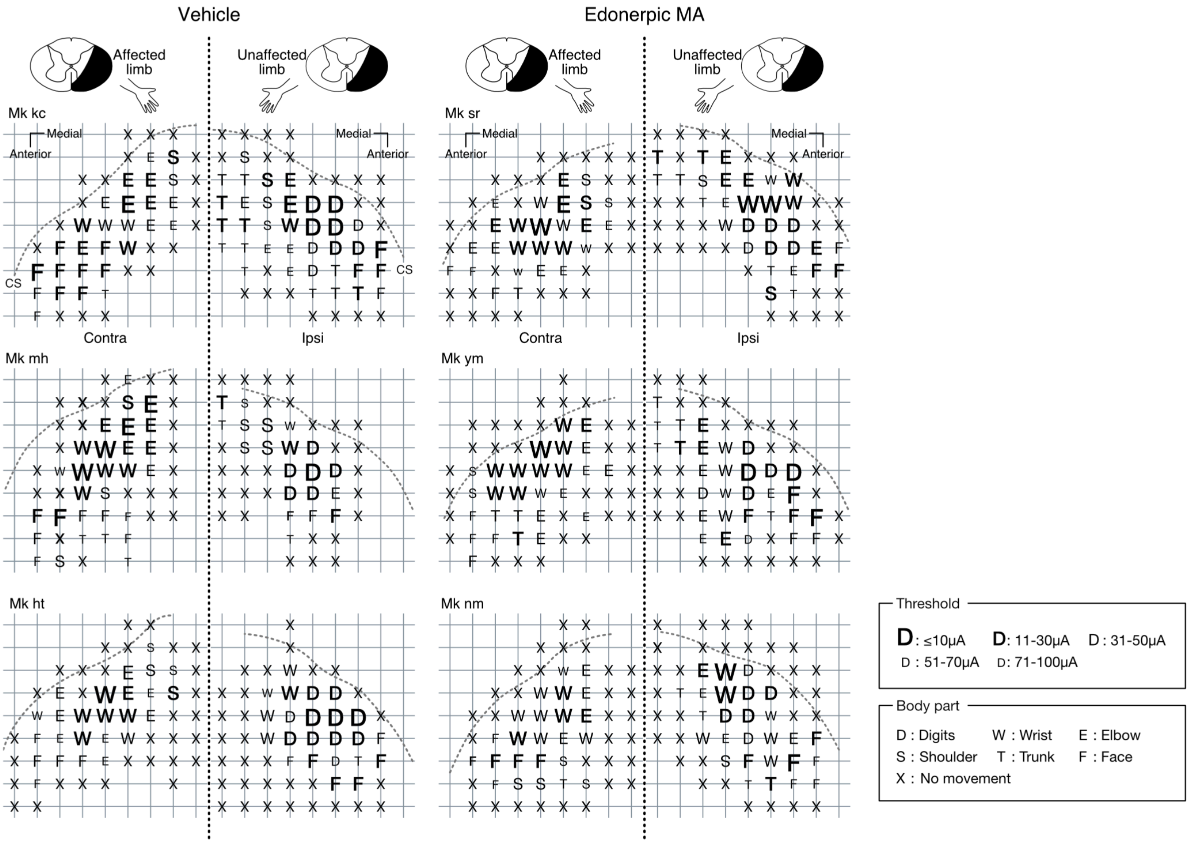

Supplementary Figure 6 Results of ICMS mapping of both sides of the hemisphere in each monkey (Results of contralesional hemisphere overlap with Figure 3). At each penetration point, the movement triggered by the lowest threshold is recorded as the body part representing that point. CS; central sulcus. Mk kc, mh, ht, sr, ym, and nm represent the identification code of the monkeys.

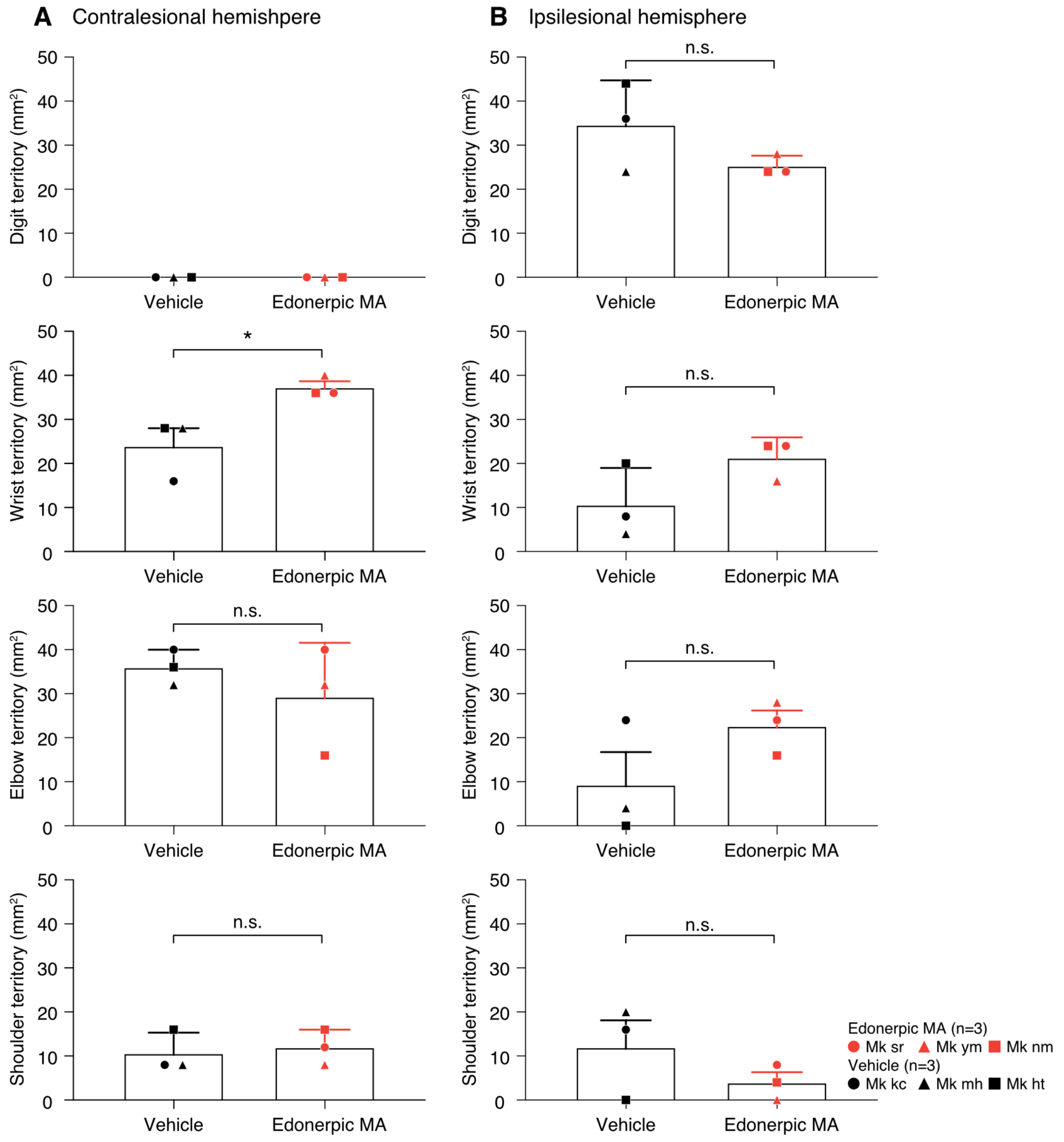

Supplementary Figure 7 Comparison of each territory on both sides of the hemisphere. (Wrist territory on the contralesional hemisphere overlaps with Figure 3). (A) There was no statistical difference between the edonerpic MA-administered monkeys (N = 3) and the vehicle-administered monkeys (N = 3) in the elbow and shoulder territory on the contralesional hemisphere (Digit; not available, Wrist; *P = 0.03, Elbow; P = 0.41, Shoulder; P = 0.72, unpaired t-test). **(B)** There was no statistical difference between the edonerpic MA-administered monkeys (N = 3) and the vehicle-administered monkeys (N = 3) in any territory on the ipsilesional hemisphere (Digit; P = 0.19, Wrist; P = 0.12, Elbow; P = 0.18, Shoulder; P = 0.29, unpaired t-test). n.s.; not statistically significant.

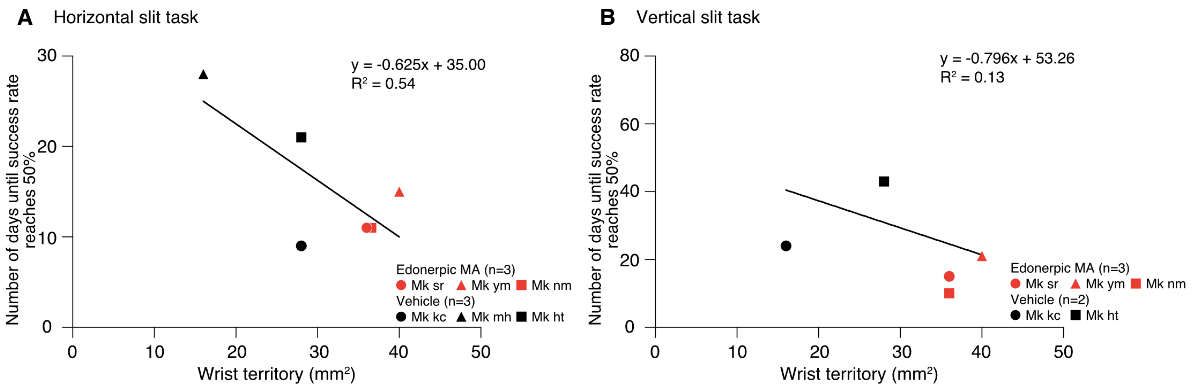

Supplementary Figure 8 Correlation between the number of days until the success rate reaches 50% and the size of wrist territory. (A) It was observed that the number of days until success rate reaches 50% in the horizontal slit task is associated with the size of wrist territory (N = 6, Pearson's product-rate correlation coefficient, $P = 0.10$). (B) In the vertical slit task, there was no statistically significant correlation between the size of the wrist territory and the number of days until the success rate reached 50% (N = 5, Pearson's product-rate correlation coefficient, $P = 0.48$). Mk mh is not included in the graph because the success rate did not reach 50%. Mk kc, mh, ht, sr, ym, and nm represent the identification code of the monkeys.
